# Draft genome sequence of a monokaryotic model brown-rot fungus *Postia (Rhodonia) placenta* SB12

**DOI:** 10.1016/j.gdata.2017.08.003

**Published:** 2017-08-10

**Authors:** Jill Gaskell, Phil Kersten, Luis F. Larrondo, Paulo Canessa, Diego Martinez, David Hibbett, Monika Schmoll, Christian P. Kubicek, Angel T. Martinez, Jagjit Yadav, Emma Master, Jon Karl Magnuson, Debbie Yaver, Randy Berka, Kathleen Lail, Cindy Chen, Kurt LaButti, Matt Nolan, Anna Lipzen, Andrea Aerts, Robert Riley, Kerrie Barry, Bernard Henrissat, Robert Blanchette, Igor V. Grigoriev, Dan Cullen

**Affiliations:** a USDA Forest Products Laboratory, Madison, WI, USA; b Millennium Nucleus for Fungal Integrative and Synthetic Biology, Pontificia Universidad Católica de Chile, Santiago, Chile; c Universidad Andres Bello, Santiago, Chile; d Los Alamos National Laboratory/Joint Genome Institute, Los Alamos, NM, USA; e Biology Department, Clark University, Worcester, MA, USA; f Austrian Institute of Technology GmbH, Vienna, Austria; g Institute of Chemical, Environmental and Biological Engineering, Vienna, Austria; h IPSBB unit, CIB, CSIC, Ramiro de Maeztu 9, E-28040, Madrid, Spain; i Department of Environmental Health, University of Cincinnati, Cincinnati, OH, USA; j Chemical Engineering, University of Toronto, Toronto, Ontario, Canada; k Pacific Northwest National Laboratory, P.O. Box 999, Richland, WA, USA; l Novozymes Inc., 1445 Drew Avenue, Davis, CA, USA; m US Department of Energy Joint Genome Institute, 2800 Mitchell Avenue, Walnut Creek, CA, USA; n Architecture et Fonction des Macromolécules Biologiques, Centre National de la Recherche, France; o Scientifique, Université d'Aix-Marseille, France; p Institut National de la Recherche Agronomique, USC 1408 AFMB, Marseille, France; q Department of Biological Sciences, King Abdulaziz University, Jeddah, Saudi Arabia; r University of Minnesota, St. Paul, MN, USA

**Keywords:** *Postia placenta*, Rhodonia placenta, Monokaryon

We report the genome of *Postia (Rhodonia) placenta* MAD-SB12, a homokaryotic wood decay fungus (Basidiomycota, Polyporales). Intensively studied as a representative brown rot decayer, the gene complement is consistent with the rapid depolymerization of cellulose but not lignin.

**Table:** 

*Specifications*	
Sex	N/A
Organism/cell line/tissue	*Postia (Rhodonia) placenta* Mad-SB12
Sequencer or array type	Illumina paired-end, 454 titanium, Sanger
Data format	Analyzed
Experimental factors	Genomic DNA from pure culture
Experimental features	Draft genome assembly and annotation
Consent	N/A
Sample source location	*Pseudotsuga menziesii*, Maryland, USA

## 1 Direct links to deposited data

The whole genome project has been deposited at DDJB/EMBL/GenBank under accession NEDQ00000000. The version described in this paper is version NEDQ00000000.1. The annotated genome is also available via the Joint Genome Institute fungal portal MycoCosm ([1]; http://genome.jgi.doe.gov/PosplRSB12_1.

## 2 Experimental design, materials and methods

Common inhabitants of forest litter and decaying wood, brown-rot fungi play a key role in carbon cycling. These Basidiomycetes rapidly depolymerize cellulose while leaving the bulk of lignin as a modified residue. The preponderance of evidence supports oxidative mechanisms involving diffusible hydroxyl radicals, but much uncertainty remains. To examine the system more closely, a dikaryotic isolate of the brown-rot fungus, *Postia placenta* (which is also classified in the genus *Rhodonia* [2]), was previously sequenced [3]. The genome has been used for phylogenomic comparisons and for analyses of transcriptomes and secretomes, but investigations are hampered by allelic variation [4], [5], [6], [7], [8], [9], [10], [11], [12], [13].

Addressing this problem, single basidiospores were collected from the fruiting dikaryon strain Mad-698 by inverting agar plates containing malt extract medium. The basidiospores were eluted from the lids with sterile water and, after streaking onto agar, individual germinating basidiospores were transferred to new plates. The monokaryotic condition was confirmed by PCR amplification and direct sequencing of genes encoding a glycosyl transferase family 66, and representatives of glycoside hydrolase families 55 and 1 [14].

The genome of *P. placenta* MAD-SB12 was sequenced using a combination of platforms: 454 (Roche), Illumina, and Sanger. Firstly, Illumina reads obtained from 300 bp insert size library sequenced in 2 × 72 bp format were assembled using Velvet [15], followed by shredding the velvet assemblies into ~ 1000 bp fragments. Then, these fragments were assembled with 454 Titanium standard and 2.8 kb insert size paired-end reads as well as Sanger fosmids using Newbler (2.5-internal-10Apr08-1) (Roche). The 42.5 Mbp genome assembly consisted of 549 scaffolds and 1446 contigs (scaffold N50 and L50 were 8 and 2.1 Mbp, respectively). Secretion signals were predicted in 1047 sequences. Assembly and general annotation features are summarized in Table 1.

**Table 1:** Assembly and annotation features of *P. placenta* SB12.

Feature	Value
Genome assembly size (Mbp)	42.45
Sequencing read coverage depth	47.36
# of contigs	1446
# of scaffolds	549
# of scaffolds ≥ 2 kbp	549
Scaffold N50	8
Scaffold L50 (Mbp)	2.10
# of gaps	897
% of scaffold length in gaps	6.1%
Three largest Scaffolds (Mbp)	4.33, 3.52, 3.23
Gene models	12,541
Average and median protein length	429, 354
Genes with Interpro domains	7221
Genes with GO terms	5937

## 3 Data description

Consistent with the degradative potential of brown rot fungi, no ligninolytic peroxidases, cellulose binding modules, or members of glycoside hydrolase (GH) families 6 and 7 were detected in the *P. placenta* SB12 genome (Table 2). Among the brown rot fungi, potential cellulases included representatives of glycoside hydrolase (GH) families GH5, GH45 and GH12. However, like the GH7s in *Laetiporus sulphureus*, none of the brown rot catalytic domains are associated with a family 1 cellulose binding module (CBM1), and their activity on crystalline cellulose is therefore suspect. In the white rot fungi, these exocellobiohydrolases and endoglucanases are typically fused to family CBM1 domains (Table 2). A total of 326 *P. placenta* SB12 genes encode carbohydrate active enzymes (CAZys), of which 144 are glycoside hydrolases [14].

**Table 2:** Genes predicted to be involved in lignocellulose degradation by wood decay fungi.

CAZy category^a^	Brown-rot^b^							White-rot^c^	
	Pospl1_SB	Pospl1	Antsi	Daequ	Fompi	Laesu	Wolco	Phach	Cersu
Auxilliary Acitivities Family AA1_1 Laccase	2	4	5	3	5	3	3	0	7
Auxilliary Acitivities Family AA1_2 Ferroxidase	1	1	1	1	1	1	1	1	1
Auxilliary Acitivities Family AA2 peroxidases	0	0	0	0	0	0	0	15	17
Auxilliary Acitivities Family AA3_1 CDH	0	0	0	0	0	0	0	1	1
Auxilliary Acitivities Family AA3_3 Alcohol oxidase	5	1	6	4	5	4	6	3	4
Auxilliary Acitivities Family AA6 BQR	1	0	1	1	1	1	1	4	0
Auxilliary Acitivities Family AA9 LPMO	2	2	2	4	4	2	2	16	9
Total AA-encoding genes	40	29	36	36	43	46	27	89	61
Carbohydrate binding modules family 1 (CBM1)	0	0	0	0	0	0	0	36	16
Total CBM-encoding genes	33	32	18	19	32	19	17	65	37
Glycoside hydrolase family GH12	2	2	3	2	2	2	2	2	2
Glycoside hydrolase family GH131	0	0	0	1	1	0	0	3	1
Glycoside hydrolase family GH133	1	0	1	1	1	1	1	1	1
Glycoside hydrolase family GH135	0	0	0	0	0	0	0	1	2
Glycoside hydrolase family GH30_3	3	0	2	3	4	1	2	1	1
Glycoside hydrolase family GH37	3	7	2	3	2	2	4	2	2
Glycoside hydrolase family GH45	1	1	0	1	2	2	0	2	2
Glycoside hydrolase family GH5_22	2	1	2	2	2	2	2	2	2
Glycoside hydrolase family GH5_31	1	0	2	3	2	0	2	1	1
Glycoside hydrolase family GH5_5	3	3	2	2	3	2	2	2	2
Glycoside hydrolase family GH51	1	2	1	1	3	2	4	2	2
Glycoside hydrolase family GH6	0	0	0	0	0	0	0	1	1
Glycoside hydrolase family GH7	0	0	0	0	0	2	0	8	3
Glycoside hydrolase family GH74	0	0	0	0	0	0	0	4	1
Glycoside hydrolase family GH78	3	1	2	3	4	3	3	1	1
Glycoside hydrolase family GH79	2	0	3	4	4	4	3	8	8
Glycoside hydrolase family GH9	0	0	0	0	0	0	0	1	0
Total GH-encoding genes	144	129	140	160	198	152	147	181	169
Total GlycosylTransferase (GT)-encoding genes	70	24	64	65	73	68	67	70	66
Total Polysaccharide Lyase (PL)-encoding genes	5	1	3	2	3	3	2	6	6
Total	326	243	285	311	388	315	288	444	369

a Abbreviations: CAZy, Carbohydrate Active Enzyme classifications [14]; CDH, Cellobiose dehydrogenase; CRO, Copper radical oxidase; BQR, Benzoquinone reductase; LPMO, Lytic polysaccharide monooxygenase.

b Brown-rot genomes: Pospl-SB12, *P. placenta* monokaryotic strain described here; Pospl1, *P. placenta* dikaryotic strain (http://genome.jgi.doe.gov/Pospl1/Pospl1.home.html); Antsi, *Antrodia sinuosa* (http://genome.jgi.doe.gov/Antsi1/Antsi1.home.html); Daequ, *Daedalea quercina* (http://genome.jgi.doe.gov/Daequ1/Daequ1.home.html); Fompi, *Fomitopsis pinicola* (http://genome.jgi.doe.gov/Fompi3/Fompi3.home.html); Laesu, *Laetiporus sulpureus* (http://genome.jgi.doe.gov/Laesu1/Laesu1.home.html); Wolco, *Wolfiporia cocos* (http://genome.jgi.doe.gov/Wolco1/Wolco1.home.html).

c White-rot genomes: Phach, *Phanerochaete chrysosporium* (http://genome.jgi.doe.gov/Phchr2/Phchr2.home.html); Cersu, *Ceriporiopsis subvermispora* (http://genome.jgi.doe.gov/Cersu1/Cersu1.home.html).

To recognize single haplotypes within the dikaryon, BLASTN alignments of putative alleles plus 500 bp of upstream regions were used to delete 4996 allelic variants. This resulted in 12,227 total gene predictions [3], an estimate similar to the actual number of haplotypes shown here in the monokaryon (12,541). However, a substantial number of genes involved in lignocellulose degradation were not captured by the computational approach. For example, dikaryotic *P. placenta* MAD-698 was predicted to encode only 243 CAZys including 129 GHs [3]. Glycosyl transferases were particularly underestimated in the dikaryon, as were 15 GHs and several oxidoreductases (Table 2). Among the latter, alcohol oxidase genes (AA3_3) are particularly important as evidence suggests their peroxide-generating activity may be directly related to the generation of small molecular weight oxidants via Fenton chemistry [16].
